# Delayed Antibody and T-Cell Response to BNT162b2 Vaccination in the Elderly, Germany

**DOI:** 10.3201/eid2708.211145

**Published:** 2021-08

**Authors:** Tatjana Schwarz, Pinkus Tober-Lau, David Hillus, Elisa T. Helbig, Lena J. Lippert, Charlotte Thibeault, Willi Koch, Irmgard Landgraf, Janine Michel, Leon Bergfeld, Daniela Niemeyer, Barbara Mühlemann, Claudia Conrad, Chantip Dang-Heine, Stefanie Kasper, Friederike Münn, Kai Kappert, Andreas Nitsche, Rudolf Tauber, Sein Schmidt, Piotr Kopankiewicz, Harald Bias, Joachim Seybold, Christof von Kalle, Terry C. Jones, Norbert Suttorp, Christian Drosten, Leif Erik Sander, Victor M. Corman, Florian Kurth

**Affiliations:** Charité–Universitätsmedizin Berlin, Berlin, Germany (T. Schwarz, P. Tober-Lau, D. Hillus, E.T. Helbig, L.J. Lippert, C. Thibeault, W. Koch, L. Bergfeld, D. Niemeyer, B. Mühlemann, C. Conrad, C. Dang-Heine, S. Kasper, F. Münn, K. Kappert, R. Tauber, S. Schmidt, P. Kopankiewicz, H. Bias, J. Seybold, C. von Kalle, T.C. Jones, N. Suttorp, C. Drosten, L.E. Sander, V.M. Corman, F. Kurth);; Hausarztpraxis am Agaplesion Bethanien Sophienhaus, Berlin (I. Landgraf);; Robert Koch Institute, Berlin (J. Michel, A. Nitsche);; German Centre for Infection Research, Berlin (D. Niemeyer, B. Mühlemann, T.C. Jones, C. Drosten, V.M. Corman);; Berlin Institute of Health, Berlin (C. Dang-Heine, K. Kappert, R. Tauber, S. Schmidt, J. Seybold, C. von Kalle);; Labor Berlin–Charité Vivantes GmbH, Berlin (K. Kappert, R. Tauber);; University of Cambridge, Cambridge, UK (T.C. Jones); G; erman Center for Lung Research, Gießen, Germany (N. Suttorp, L.E. Sander, F. Kurth);; Bernhard Nocht Institute for Tropical Medicine, Hamburg, Germany (F. Kurth);; University Medical Centre Hamburg-Eppendorf, Hamburg (F. Kurth)

**Keywords:** vaccine, mRNA, immunity, T cell, B cell, antibody, COVID-19, coronavirus disease, SARS-CoV-2, severe acute respiratory syndrome coronavirus 2, viruses, respiratory infections, zoonoses, Berlin, Germany

## Abstract

We detected delayed and reduced antibody and T-cell responses after BNT162b2 vaccination in 71 elderly persons (median age 81 years) compared with 123 healthcare workers (median age 34 years) in Germany. These data emphasize that nonpharmaceutical interventions for coronavirus disease remain crucial and that additional immunizations for the elderly might become necessary.

The severe acute respiratory syndrome coronavirus 2 (SARS-CoV-2) pandemic has led to an urgent need for vaccines, particularly among persons at high risk for severe disease and death, such as the elderly (*1*). Efficacy against severe coronavirus disease (COVID-19) of mRNA vaccine BNT162b2 (Pfizer-BioNTech, https://www.pfizer.com) is reported to be >90% starting 7 days after the second vaccination; robust antibody and T-cell response has been demonstrated consistently across age groups (*2*–*4*). However, only 4.3% of participants in the BNT162b2 efficacy trial were >75 years of age (*4*). Given the elderly generally have weaker immune responses after vaccination, more detailed investigation is necessary (*4*,*5*).

## The Study

In a prospective observational cohort study, we investigated SARS-CoV-2–specific antibodies, maturation of IgG avidity, and interferon-γ (IFN-γ) release of SARS-CoV-2–specific T cells in 2 cohorts of young and elderly BNT162b2-vaccinated persons (Table). Participants were recruited from 2 studies conducted at Charité–Universitätsmedizin Berlin, both conducted in accordance with the Declaration of Helsinki and Good Clinical Practice (https://www.ema.europa.eu/en/documents/scientific-guideline/ich-e-6-r2-guideline-good-clinical-practice-step-5_en.pdf) and approved by the local ethics committee (EA4/244/20 and EA4/245/20)

**Table:** Cohort characteristics in study of delayed antibody and T-cell response to BNT162b2 vaccination in the elderly, Germany*

Characteristic	Healthcare workers		Elderly	
No. patients	123		71	
Sex				
F	65 (52.9)		54 (76.1)	
M	58 (47.2)		17 (23.9)	
Median age, y (IQR)	34 (20–64)		81 (70–96)	
Underlying conditions				
Cardiovascular disease	15 (12.2)		56 (78.9)	
Type 2 diabetes	1 (0.8)		13 (18.3)	
Respiratory disease	16 (13.0)		11 (15.5)	
Dyslipidemia	5 (4.1)		21 (29.6)	
Thyroid dysfunction	0		16 (22.5)	
Chronic kidney disease	0		12 (16.9)	
Chronic liver or GI disease	2 (1.6)		18 (25.4)	
Rheumatic disease	6 (4.9)		7 (9.9)	
Active solid malignancy	2 (1.6)		4 (5.6)	
Active hematological malignancy	0		4 (5.6)	
Neurologic disease	1 (0.8)		18 (25.4)	
Immunodeficiency	1 (0.8)		0	
Others	9 (7.3)		29 (40.9)	
Outpatient medication				
No	79 (64.2)		5 (7.0)	
Yes	39 (31.7)		64 (90.1)	
Unknown	5 (4.1)		2 (2.8)	

*Values are no. (%) except as indicated. BNT162b2 is manufactured by Pfizer-BioNTech (https://www.pfizer.com). GI, gastrointestinal; IQR, interquartile range.

The first cohort consisted of 123 healthcare workers; median age was 34 (interquartile range [IQR] 20–64) years. The second cohort consisted of 71 elderly residents of an assisted living facility; median age was 81 (IQR 70–96) years. Blood samples were taken before the first vaccination (week 0), just before the second vaccination (week 3), and 4 weeks after the second vaccination (week 7). To discriminate between vaccine-induced antibody response and convalescent SARS-CoV-2 infection, we used the SeraSpot Anti-SARS-CoV-2 IgG microarray-based immunoassay including nucleocapsid and spike as antigens (Seramun Diagnostica GmbH, https://www.seramun.com) (Appendix). Ten of 123 healthcare workers and 1 of 71 elderly participants showed reactive anti-nucleocapsid or anti-spike IgG before the first vaccination and were excluded from further analyses.

At week 3, in the younger cohort, 93/107 (86.9%, 95% CI 79.2%–92.0%) participants showed reactive SARS-CoV-2 receptor-binding domain (RBD) IgG, compared with only 16/52 elderly participants (30.8%, 95% CI 19.9%–44.3%). At week 7, the antibody response rate had increased in both cohorts, to 112/113 in younger participants (99.1%, 95% CI 95.2%–100.0%) and 64/70 in the elderly cohort (91.4%, 95% CI 82.5%–96.0%) (Figure, panel A; Appendix Table). The comparison of SARS-CoV-2 RBD IgG levels demonstrated a significant difference in the 2 cohorts at both week 3 (p<0.0001) and week 7 (p = 0.0003) (Appendix Table), indicating a substantial delay and overall reduced antibody response in elderly participants. We observed similar kinetics and differences between cohorts for antibody responses to 2 further SARS-CoV-2 spike antigens: the S1 subdomain and the full spike protein (Appendix Table, Figure).

**Figure:**
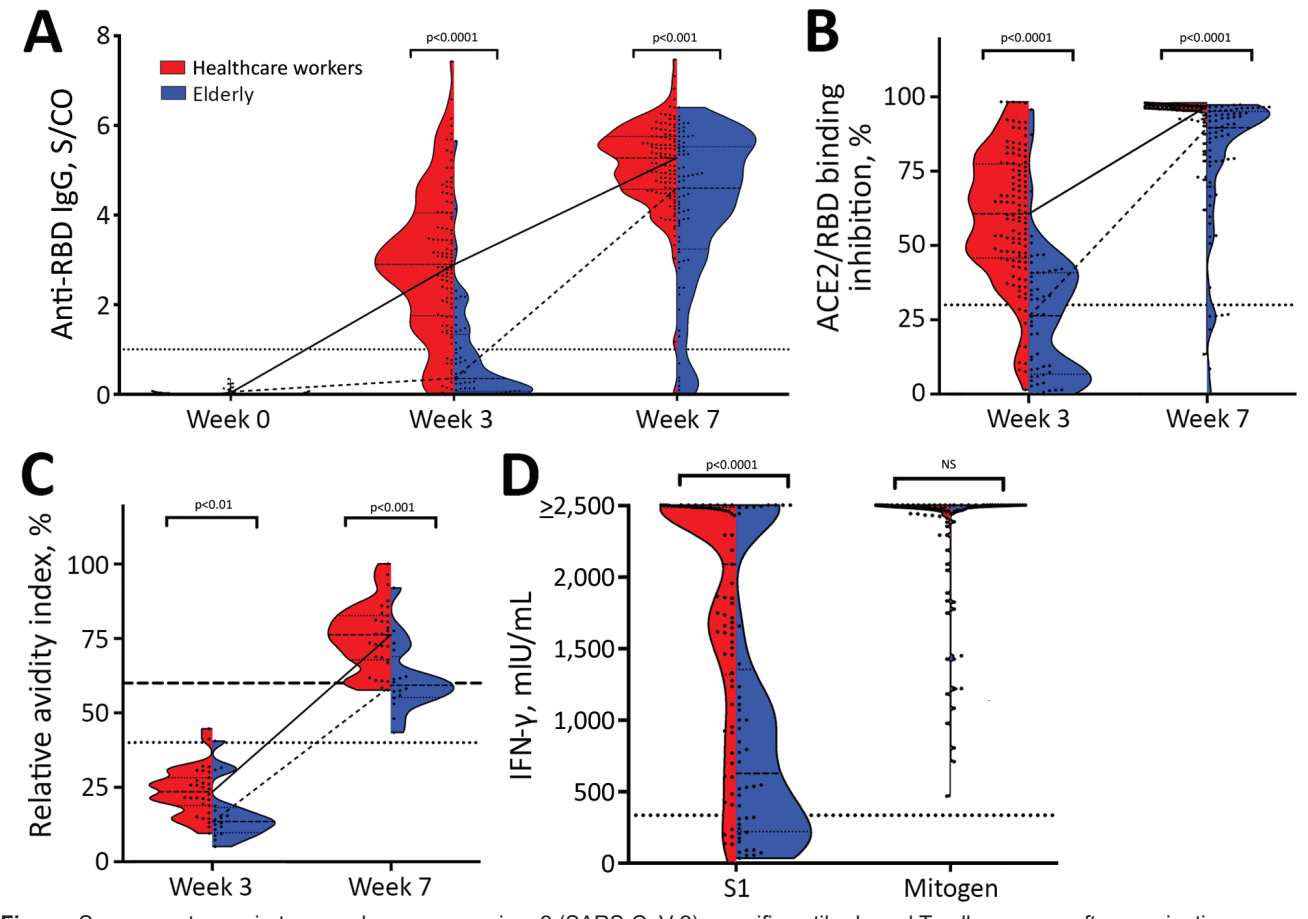
Severe acute respiratory syndrome coronavirus 2 (SARS-CoV-2)–specific antibody and T-cell response after vaccination with BNT162b2 (Pfizer-BioNTech, https://www.pfizer.com) in the elderly, Germany. A) SARS-CoV-2 RBD IgG measured in serum of BNT162b2-vaccinated younger participants (healthcare workers) before the first vaccination (n = 100, week 0), 3 weeks after the first vaccination (n = 107, week 3), and 4 weeks after the second vaccination (n = 113, week 7) and from elderly participants at week 0 (n = 70), week 3 (n = 52), and week 7 (n = 70) using the SeraSpot Anti-SARS-CoV-2 IgG assay (Seramun Diagnostica GmbH, https://www.seramun.com). B) Neutralizing capacity of antibodies measured at week 3 and 7 in the young and elderly cohorts using the ELISA-based surrogate virus neutralization test (sVNT) cPass (medac GmbH, https://international.medac.de). C) SARS-CoV-2 spike IgG avidity analyzed in the healthcare workers cohort (n = 30) and elderly cohort (n = 16) at week 3 and 7. D) At week 7, whole blood from vaccinated elderly participants (n = 43) and young participants (n = 71) was stimulated ex vivo with components of the S1 domain of the spike protein for 24 h, and IFN-γ concentration in the supernatant was detected by ELISA. Dotted lines indicate the manufacturer’s specified threshold for RBD IgG >1 S/Co, for sVNT >30%, and for avidity 40–60% borderline avidity and >60% high avidity. For IGRA, we defined an arbitrary threshold at 334.2 mIU/mL. p value was calculated by the nonparametric Mann Whitney U test, and the median and interquartile range are depicted. ACE2, angiotensin-converting enzyme 2; IFN-γ, interferon-γ; IU, international units; NS, not significant; RBD, receptor-binding domain; S/CO, signal-to-cutoff ratio; sVNT, surrogate virus neutralization test.

We further confirmed the delayed and reduced antibody response in the elderly by measurement of the functional neutralization capacity using the surrogate virus neutralization test (sVNT) cPass (medac GmbH, https://international.medac.de) (Appendix) (*6*). At week 3, only 24/52 elderly participants (46.2%, 95% CI 33.3%–59.5%) had neutralizing capacity in serum, compared with 97/107 younger participants (90.7%, 95% CI 83.7%–94.8%; p<0.0001 (Figure). In addition, the median sVNT titer for elderly participants was significantly lower than the young cohort (26.4% [IQR 6.8%–40.9%] vs. 60.2% [IQR 45.0%–76.4%]; p<0.0001) (Appendix Table). Although the neutralizing antibody response rate increased to 63/70 (90.0%, 95% CI 80.8%–95.1%) in elderly participants by week 7, the median sVNT titer remained significantly lower than in the younger cohort (89.6% [IQR 70.9%–95.2%] vs. 96.7% [IQR 95.6%–97.2%]; p<0.0001) (Appendix Table).

To characterize the maturation of IgG avidity in all 16 elderly and 30 randomly selected younger participants who were seroreactive at week 3, we applied a modified SARS-CoV-2 S1 IgG ELISA (Euroimmun, https://www.euroimmun.com) (Appendix Figure 2). The delayed antibody response in the elderly is reflected in results: at week 7, only 8/16 elderly (50.0%, 95% CI 28.0%–72.0%) exhibited high S1 IgG avidity indices (>60) compared with 28/30 young participants (93.3%, 95% CI 78.7%–98.8%) (Figure, panel C). Consequently, the median relative avidity index of IgG was significantly higher in the younger cohort than the elderly cohort (76.2% [IQR 67.6%–82.9%] vs. 59.3% [IQR 55.3%–68.9%]; p = 0.0002) (Figure, panel C).

In addition to antibody responses, we assessed SARS-CoV-2 spike-specific T cell responses by an IFN-γ release assay (IGRA) (Euroimmun) (Appendix) of S1 peptide-stimulated T cells at week 7. The proportion of persons with IGRA results above the defined threshold (Appendix) was significantly lower in the elderly than in younger participants (51.2% [95% CI 36.8%–65.4%] vs. 84.5% [95% CI 74.4%–91.1%]; p = 0.0002). Accordingly, median S1-induced IFN-γ release was significantly decreased in the elderly compared to younger participants (707.3 mIU/mL [IQR 216–1,392] vs. 2184 mIU/mL [IQR 1,274–2,484]; p<0.0001) (Figure, panel D). In contrast, we detected no significant difference in IFN-γ release after mitogen stimulation between the 2 cohorts, indicating no general impairment of IFN-γ responses in the elderly (p = 0.77) (Figure, panel D; Appendix Table).

In summary, vaccination with BNT162b2 induces both arms of adaptive immunity: SARS-CoV-2–specific antibodies and SARS-CoV-2–specific T cells. However, we observed delayed and less robust cellular and humoral immune response among the elderly than among younger adults. A limitation of our study is the lack of data on other COVID-19 vaccines. Furthermore, we cannot exclude that underlying diseases or medications, which are more common in the elderly (Table), might impair the vaccine-induced immune response. For example, patients on dialysis have significantly lower antibody response than vaccinated same-age patients not on dialysis (E. Schrezenmeier et al., unpub. data, http://medrxiv.org/lookup/doi/10.1101/2021.03.31.21254683).

Our data are supported by other real-world observations suggesting a delayed and reduced immunogenicity of BNT162b2 in the elderly (*5**,**7*; D.A. Collier et al., unpub. data, http://medrxiv.org/lookup/doi/10.1101/2021.02.03.21251054). In line with our observations for BNT162b2, an effect of age-dependent decrease of immune function, referred to as immunosenescence, is well known and contributes to increased prevalence of infectious disease and vaccine failure in the elderly (*8*). A lower vaccine-induced immune response to influenza and hepatitis B viruses is well documented (*9*,*10*); however, such data are scarce for mRNA vaccines.

Of note, vaccination with 2 doses of BNT162b2 might not fully prevent SARS-CoV-2 outbreaks among elderly persons in congregate settings, such as long-term care facilities, possibly because of delayed and reduced immune response. However, vaccination protects against severe disease (*11*–*13*).

## Conclusions

Although the immune response of elderly participants 4 weeks after the second dose of BNT162b2 nearly reached the level of younger participants, a small fraction of elderly participants did not demonstrate robust antibody and T-cell response. However, the immunologic correlates of protection remain unknown, and identification of persons with no or incomplete protection after vaccination remains challenging. Therefore, strategies focused solely on vaccinating of high-risk groups might be insufficient to protect those at risk for severe disease. For the elderly, vaccination of caregivers and close contacts should be prioritized. Moreover, a booster vaccination, altered vaccine dose, or different COVID-19 vaccines should be considered for the elderly if further evidence demonstrates high rates of breakthrough infections despite 2-dose BNT162b2 vaccination.

These results are particularly relevant for vaccination strategies focused on broad administration of the first dose of a 2-dose vaccine while postponing the second vaccination. This practice might leave a relevant proportion of elderly with comparatively low levels of immunity for a prolonged period, emphasizing the need for nonpharmaceutical interventions, such as mask use and regular testing.

Appendix Additional information about delayed antibody and T-cell response to BNT162b2 vaccination in the elderly, Germany
