# Supplementary material for: Delayed Antibody and T-Cell Response to BNT162b2 Vaccination in the Elderly, Germany
Source: Emerg Infect Dis. 2021 Aug;27(8):2174–8. doi: 10.3201/eid2708.211145 (PMC8314803; doi:10.3201/eid2708.211145)
Supplement: Appendix — Additional information about delayed antibody and T-cell response to BNT162b2 vaccination in the elderly, Germany [file 21-1145-Techapp-s1.pdf]

Article DOI: https://doi.org/10.3201/eid2708.211145

Delayed Antibody and T-Cell Response to BNT162b2 Vaccination in the Elderly, Germany

Appendix

Patients, Materials and Methods

Elderly Cohort

Patients were recruited within the COVIMMUNIZE study conducted under the auspices of Charité–Universitätsmedizin Berlin, a prospective observational cohort study on immunogenicity of coronavirus disease (COVID-19) vaccines in populations at risk. Written informed consent was obtained from patients or legal representatives according to regulations set by the ethics committee of Charité–Universitätsmedizin Berlin.

Subjects were eligible for inclusion if they were >70 years of age and scheduled for vaccination against COVID-19 with BNT162b2. To detect concurrent and asymptomatic severe acute respiratory syndrome coronavirus 2 (SARS-CoV-2) infections, weekly reverse transcription PCR (RT-PCR) of oropharyngeal swab specimens was conducted for the first 8 weeks after enrollment. Blood sampling was performed immediately before the first (week 0) and second (week 3) dose of vaccine, and at week 7–8 (4 weeks after the second vaccination). Antibody and T cell data obtained from week 7 samples from 44 participants have been used as a control cohort in another study (E. Schrezenmeier et al., unpub. data, http://medrxiv.org/lookup/doi/10.1101/2021.03.31.21254683).

Healthcare Workers Cohort

Healthcare workers of the Charité–Universitätsmedizin Berlin were recruited within the EICOV study, a prospective observational cohort study on immunogenicity and reactogenicity of COVID-19 vaccines in healthcare workers. Subjects were eligible for inclusion if they were >18 years of age, employed at the Charité–Universitätsmedizin Berlin, and had no contraindication against a COVID-19 vaccination. To detect concurrent and asymptomatic infections with SARS-

CoV-2, weekly self-collected oropharyngeal swab specimens from participants were analyzed after enrollment. Blood sampling took place immediately before the first (week 0) and second (week 3) dose of vaccination with BNT162b2, as well as at week 7–8 (4 weeks after the second vaccination). Antibody and T cell data obtained from the 123 healthcare workers have been used as a control cohort in another study (D. Hillus et al., unpub. data, https://www.medrxiv.org/content/10.1101/2021.05.19.21257334v2).

Antibody Assessment

For detection of SARS-CoV-2–specific antibodies to the spike and nucleocapsid proteins, we used the SeraSpot Anti-SARS-CoV-2 IgG microarray-based multiparameter immunoassay according to manufacturer's instructions (Seramun Diagnostica GmbH, https://www.seramun.com). In brief, this assay is based on the use of 4 recombinant SARS-CoV-2 proteins (complete spike, S1 domain, receptor-binding domain [RBD], and nucleocapsid) as capture antigens. These and test-specific controls are printed in an array arrangement on the bottom of each well. Bound antibodies from the patient serum samples are detected by horseradish peroxidase–labeled antibodies against human IgG. Color intensity at the site of formed immune complexes (pale blue to dark blue) correlates with antibody concentration. The SpotSight plate scanner was used for measurements. Results are calculated and normalized as signal-to-cutoff (S/CO) ratios by dividing the observed signal strength of a specific location by that of an internal cutoff control. Samples with an S/CO ratio $\geq$1.0 are defined as positive by the manufacturer.

Surrogate SARS-CoV-2 Neutralization Test

To detect neutralizing activity in serum samples 3 weeks after the first vaccination and 4 weeks after the second vaccination, we used the commercially available ELISA-based SARS-CoV-2 surrogate neutralization test cPass (medac GmbH, https://international.medac.de) according to the manufacturer's instructions. Serum samples and positive and negative controls were diluted 1:10 in sample dilution buffer and preincubated 1:1 with RBD–horseradish peroxidase for 30 min at 37°C. Each reaction mixture was then added to the hACE2 precoated plate and incubated at 37°C for 15 min. After a washing step, 3′3,5,5-tetramethylbenzidine solution was added to each well and the plate was incubated at room temperature for 15 min. Following a stop solution step, the optical density (OD) at 450 nm was detected. Data were interpreted by the calculation of the relative inhibition using the following equation: inhibition

[%] = (1–OD value of sample/OD value of negative control) × 100. Samples were considered negative at an inhibition of <30% and considered positive otherwise.

IgG Avidity Assay

Maturation of IgG avidity was detected in younger (n = 30) and elderly (n = 16) participants at week 3 and week 7 by using an anti-SARS-CoV-2 S1 IgG ELISA Kit (Euroimmun, https://www.euroimmun.com), which showed a strong correlation with the SeraSpot Anti-SARS-CoV-2 IgG RBD ($p < 0.001$, Spearman's rank correlation coefficients = 0.662) used for general antibody testing (Appendix Figure 2). Serum samples were diluted 1:101 with sample buffer and incubated on plates precoated with recombinant SARS-CoV-2 spike proteins. After incubation for 1h at 37°C, wells were washed and 200 µL urea or 200 µL PBS were added to the plates and incubated for 10 min at 37°C. After a washing step, conjugate and substrate were added according to the manufacturer's instructions. OD was detected at 450 nm, and the relative avidity index was calculated by dividing the observed OD of the urea-treated sample by that of the PBS-treated sample, multiplied by 100. Values with an index of more than 60% were considered to be high avidity.

Interferon-γ Release of SARS-CoV-2–Specific T Cells

We applied a commercially available IGRA for assessment of interferon-γ (IFN-γ) release of SARS-CoV-2–specific T cells (Euroimmun). In parallel, 0.5 mL freshly collected Lithium-heparin blood was stimulated with a SARS-CoV-2 peptide pool from the spike S1 domain, 0.5 mL of blood was stimulated with mitogen as a positive control, and 0.5 ml of blood in a blank was used as a negative control. After 24 hours of incubation at 37°C, IFN-γ concentration in the plasma fraction of all 3 stimulation tubes was measured by ELISA. IFN-γ response in the blank served as a measure of patient-individual background IFN-γ activity and was subtracted from the IFN-γ response in the stimulation tubes. For analyses of IGRA outcome, we defined an arbitrary cutoff by using average IFN-γ activity (33.42 mIU/mL) determined in the 15 SARS-CoV-2 IgG-negative unvaccinated control multiplied by 5 as the threshold (167.1 mIU/mL) for borderline IGRA–reactive and multiplied by 10 for the threshold (334.2 mIU/mL) for IGRA–positive.

Statistical Analysis

Values are given as medians with interquartile range unless stated otherwise. GraphPad PRISM statistics version 27.0 (IBM Deutschland, https://www.ibm.com/de-de) was used for statistical analysis. Group differences were assessed in a univariate analysis by using Fisher exact test or nonparametric Mann Whitney U test. P values of <0.05 were considered statistically significant. All 95% CI for proportions were calculated by using the Wilson procedure with a correction for continuity (*1*).

References

1. Newcombe RG. Two-sided confidence intervals for the single proportion: comparison of seven methods. Stat Med. 1998;17:857–72. PubMed https://doi.org/10.1002/(SICI)1097-0258(19980430)17:8<857::AID-SIM777>3.0.CO;2-E

Appendix Table. Proportion of positive outcome and outcome values in different test systems in study of delayed antibody and T-cell response to BNT162b2 vaccination in the elderly, Germany

		Proportion of positive outcome (95% CI)			Outcome values median [IQR]		
Time	Test system	Healthcare workers	Elderly	p value*	Healthcare workers	Elderly	p value†
Week 7	NP spot array, S/CO	0.0 (0.0–3.3) [0/113]	0.0 (0.0–5.2) [0/70]		0.1 [0.1–0.2]	0.1 [0.0–0.1]	
	RBD spot array, S/CO	99.1 (95.2–100.0) [112/113]	91.4 (82.5–96.0) [64/70]	0.013	5.3 [4.6–5.8]	4.6 [3.2–5.5]	0.0003
	S1 spot array, S/CO	98.2 (93.8–99.7) [111/113]	90.0 (80.8–95.1) [63/70]	0.028	4.4 [3.9–4.89]	3.14 [2.07–4.26]	<0.0001
	Spike full spot array, S/CO	98.23 (93.78–99.69) [111/113]	91.43 (82.53–96.01) [64/70]	0.056	4.68 [4.19–5.3]	3.9 [2.8–5.0]	<0.0001
	ACE2/RBD inhibition, %	99.1 (95.2–100.0) [112/113]	90.0 (80.8–95.1) [63/70]	0.005	96.7 [95.6–97.2]	89.6 [70.9–95.2]	<0.0001
	IgG RAI, %	93.3 (78.7–98.8) [28/30]	50.0 (28.0–72.0) [8/16]	0.0015	76.2 [67.6–82.9]	59.3 [55.3–68.9]	0.0002
	IGRA S1 stimulation, mIU/mL	84.5 (74.4–91.1) [60/71]	51.2 (36.8–65.4) [22/43]	0.0002	2184 [1274–2484]	707.3 [215.5–1392]	<0.0001
	IGRA mitogen stimulation, mIU/mL	100.0 (94.9–100.0) [71/71]	100.0 (91.8–100.0) [43/43]		2483 [2438–2497]	2498 [2456–2500]	0.077
Week 3	NP spot array, S/CO	0.0 (0.0–3.5) [0/107]	0.0 (0.0–6.9) [0/52]		0.1 [0.1–0.2]	0.2 [0.1–0.2]	
	RBD spot array, S/CO	86.9 (79.2–92.0) [93/107]	30.8 (19.9–44.3) [16/52]	<0.0001	2.9 [1.8–4.1]	0.4 [0.1–1.3]	<0.0001
	S1 spot array, S/CO	66.4 (57.0–74.6) [71/107]	7.7 (3.0–18.2) [4/52]	<0.0001	1.3 [0.7–2.2]	0.2 [0.0–0.5]	<0.0001
	Spike full spot array, S/CO	84.1 (76.0–89.8) [90/107]	34.6 (23.2–48.2) [18/52]	<0.0001	2.1 [1.4–3.2]	0.4 [0.1–1.3]	<0.0001
	ACE2/RBD inhibition, %	90.7 (83.7–94.8) [97/107]	46.2 (33.3–59.5) [24/52]	<0.0001	60.2 [45.0–76.4]	26.4 [6.8–40.9]	<0.0001
	IgG RAI, %	0.0 (0.0–11.4) [0/30]	0.0 (0.0–19.4) [0/16]		23.6 [18.2–28.7]	13.6 [9.8–18.3]	0.0033
Week 0	NP spot array, S/CO	0.0 (0.0–3.7) [0/100]	0.0 [0.0–5.2) [0/70]		0.1 [0.1–0.1]	0.1 [0.1–0.2]	
	RBD spot array, S/CO	0.0 (0.0–3.7) [0/100]	0.0 [0.0–5.2) [0/70]		0.0 [0.0–0.0]	0.0 [0.0–0.0]	
	S1 spot array, S/CO	0.0 (0.0–3.7) [0/100]	0.0 [0.0–5.2) [0/70]		0.0 [0.0–0.0]	0.0 [0.0–0.0]	
	Spike full spot array, S/CO	0.0 (0.0–3.7) [0/100]	0.0 [0.0–5.2) [0/70]		0.0 [0.0–0.0]	0.0 [0.0–0.0]	

*p value was calculated by Fisher exact test. ACE2, angiotensin-converting enzyme 2; IGRA, interferon-γ release assay; IU, international units; NP, nucleocapsid protein; RAI, relative avidity index; RBD, receptor-binding domain; S1, spike subdomain 1; S/CO, signal-to-cutoff ratio.
†p value was calculated by the nonparametric Mann Whitney U test.

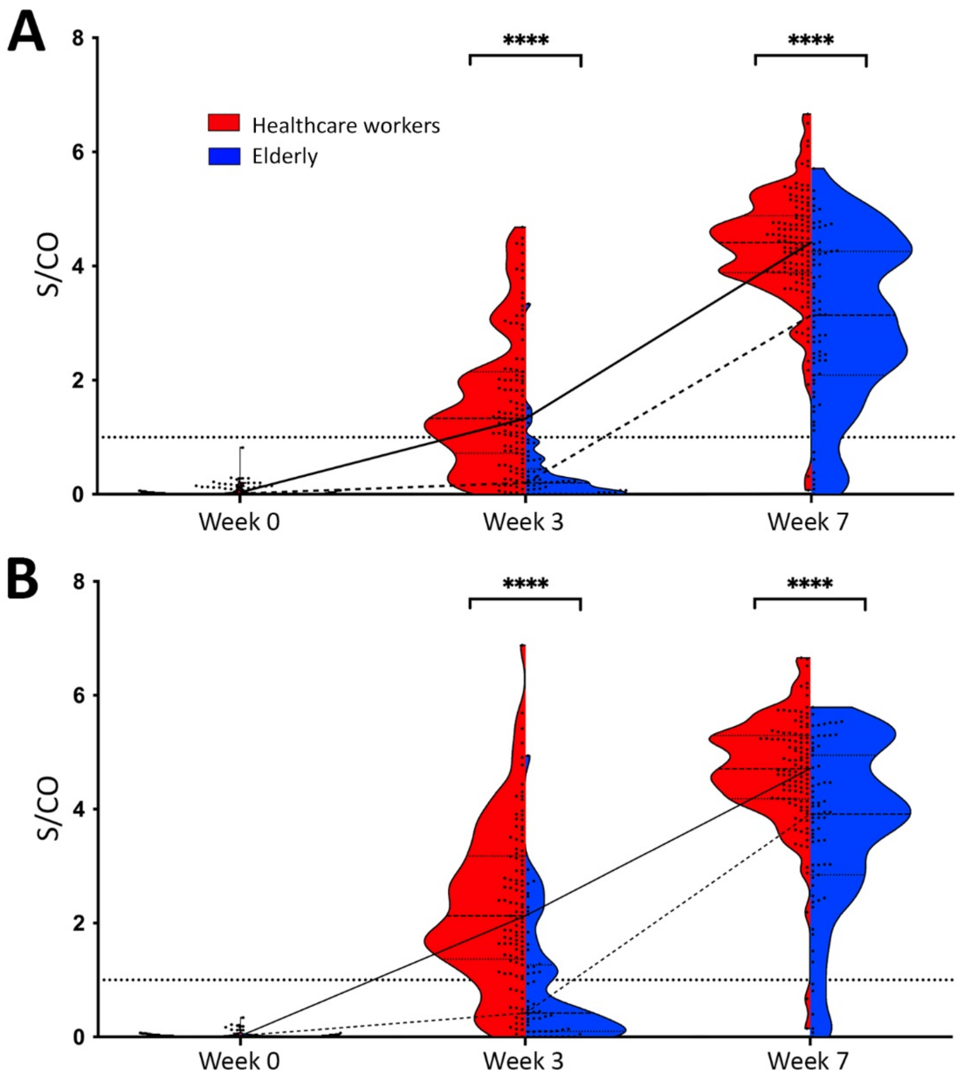

Appendix Figure 1. Anti-SARS-CoV-2 S1 and full spike IgG antibody response after BNT162b2 vaccination in the elderly, Germany. A) Anti-SARS-CoV-2 S1 and B) full spike IgG were measured in serum of BNT162b2-vaccinated healthcare workers (median age 34 years) (red) before the first vaccination (n = 100, week 0), 3 weeks after the first vaccination (n = 107, week 3), and 4 weeks after the second vaccination (n = 113, week 7) and in elderly participants (median age 81 years) (blue) at week 0 (n = 70), week 3 (n = 52), and week 7 (n = 70) using the SeraSpot Anti-SARS-CoV-2 IgG assay. Samples with an S/CO ratio of $\geq$1.0 are defined by the manufacturer as positive. P value was calculated by the nonparametric Mann Whitney U test and the median and interquartile range are depicted. S/CO, signal-to-cutoff ratio; ****, p<0.0001.

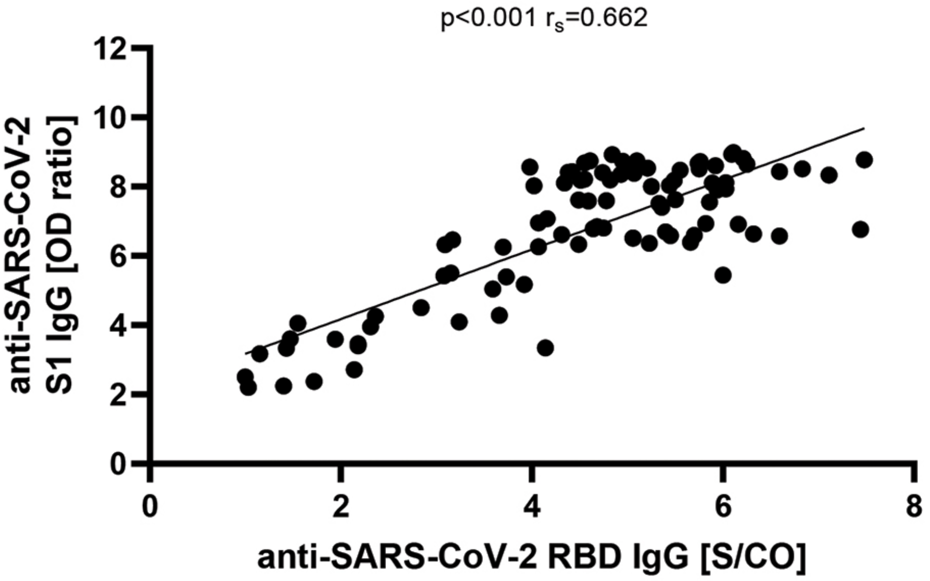

Appendix Figure 2. Correlation between anti-S1 IgG and anti-RBD IgG in study of delayed antibody and T-cell response to BNT162b2 vaccination in the elderly, Germany. Anti-SARS-CoV-2 S1 IgG OD ratios of 92 samples were correlated with anti-SARS-CoV-2 RBD IgG S/CO measurements (p value, Spearman's rank correlation coefficients). OD, optical density; RBD, receptor-binding domain; S/CO, signal-to-cutoff ratio.
